# Epigenetic age acceleration is associated with blood lipid levels in a multi-ancestry sample of older U.S. adults

**DOI:** 10.21203/rs.3.rs-3934965/v1

**Published:** 2024-02-27

**Authors:** Lisha Lin, Jenna Kiryakos, Farah Ammous, Scott M. Ratliff, Erin B. Ware, Jessica D. Faul, Sharon L.R. Kardia, Wei Zhao, Kira S. Birditt, Jennifer A. Smith

**Affiliations:** Department of Epidemiology, School of Public Health, University of Michigan; Department of Epidemiology, School of Public Health, University of Michigan; Department of Epidemiology, School of Public Health, University of Michigan; Department of Epidemiology, School of Public Health, University of Michigan; Survey Research Center, Institute for Social Research, University of Michigan; Survey Research Center, Institute for Social Research, University of Michigan; Department of Epidemiology, School of Public Health, University of Michigan; Department of Epidemiology, School of Public Health, University of Michigan; Survey Research Center, Institute for Social Research, University of Michigan; Department of Epidemiology, School of Public Health, University of Michigan

**Keywords:** Epigenetic age acceleration, DNA methylation, lipids, cholesterol, HDL cholesterol, LDL cholesterol, triglycerides, aging

## Abstract

**Background:**

Dyslipidemia, which is characterized by an unfavorable lipid profile, is a key risk factor for cardiovascular disease (CVD). Understanding the relationships between epigenetic aging and lipid levels may help guide early prevention and treatment efforts for dyslipidemia.

**Methods:**

We used weighted linear regression to cross-sectionally investigate the associations between five measures of epigenetic age acceleration estimated from whole blood DNA methylation (HorvathAge Acceleration, HannumAge Acceleration, PhenoAge Acceleration, GrimAge Acceleration, and DunedinPACE) and four blood lipid measures (total cholesterol (TC), LDL-C, HDL-C, and triglycerides (TG)) in 3,813 participants (mean age = 70 years) from the Health and Retirement Study (HRS). As a sensitivity analysis, we examined the same associations in participants who fasted prior to the blood draw (n = and f) and in participants who did not take lipid-lowering medication (n = 1,869). Using interaction models, we also examined whether the relationships between epigenetic age acceleration and blood lipids differ by demographic factors including age, sex, and educational attainment.

**Results:**

After adjusting for age, race/ethnicity, sex, fasting status, and lipid-lowering medication use, greater epigenetic age acceleration was associated with lower TC, HDL-C, and LDL-C, and higher TG (p < 0.05). GrimAge acceleration and DunedinPACE associations with all lipids remained significant after further adjusting for body mass index, smoking status, and educational attainment. These associations were stronger in participants who fasted and who did not use lipid-lowering medication, particularly for LDL-C. We observed the largest number of interactions between DunedinPACE and demographic factors, where the associations with lipids were stronger in younger participants, females, and those with higher educational attainment.

**Conclusion:**

Epigenetic age acceleration, a powerful biomarker of cellular aging, is highly associated with blood lipid levels in older adults. A greater understanding of how these associations differ across demographic groups can help shed light on the relationships between aging and downstream cardiovascular diseases. The inverse associations between epigenetic age and TC and LDL-C could be due to sample limitations or the non-linear relationship between age and these lipids, as both TC and LDL-C decrease faster at older ages. More studies are needed to further understand the temporal relationships between epigenetic age acceleration on blood lipids and other health outcomes.

## Introduction

Cardiovascular diseases (CVD) are the main causes of morbidity and mortality among U.S. adults (1). Dyslipidemia, a major risk factor for CVD (2–4), is a modifiable condition characterized by decreased high-density lipoprotein-cholesterol (HDL-C), elevated low-density lipoprotein-cholesterol (LDL-C), and/or elevated triglycerides (TG) (5). LDL-C makes up the majority of cholesterol in the human body and is associated with a higher risk of CVD (6–8), while higher HDL-C is protective for CVD (2, 6, 9). Elevated TG level is also a risk factor for CVD independent of lower HDL-C levels (10, 11). Total cholesterol (TC) is the sum of HDL-C, LDL-C, and 20% of TG (12). In the U.S., approximately 38% of adults have high TC (≥ 200 mg/dL) (13), and over 60% of adults aged over 65 years have dyslipidemia (14). Therefore, it is essential to establish further strategies for earlier prevention and treatment of dyslipidemia to reduce subsequent CVD risk. Multiple risk factors are associated with the development of dyslipidemia, including behaviors such as unhealthy diet, lack of exercise (15), and genetic factors (16–19). Epigenetic factors, such as DNA methylation, have also been associated with CVD (20–22), but it is unclear whether changes in the epigenome are precursors or consequences of CVD-related pathologies.

DNA methylation is an epigenetic mechanism that helps regulate transcription without changing the DNA sequence (23). DNA methylation happens at CpG sites, which involves the covalent addition of a methyl group to the cytosine base. Recently, there has been a surge of epigenetic research examining the relationships between biological aging and disease risk or progression (24–26). Epigenetic clocks, a method of capturing biological aging by assessing DNA methylation, differ among individuals of the same chronological age. This may be due in part to exogenous stressors such as neighborhood environment and socioeconomic status (27, 28). Epigenetic age acceleration represents the relative difference between one’s chronological age and epigenetic age, where positive values of epigenetic age acceleration indicate faster biological aging.

Five widely used epigenetic clocks include HorvathAge (29), HannumAge (30), PhenoAge (31), and GrimAge (32), which all aim to assess biological age in years, as well as DunedinPACE, which assesses the pace of aging (33). Compared with the earlier developed clocks (i.e., HorvathAge and HannumAge), the more recently developed clocks (i.e., PhenoAge, GrimAge,and DunedinPACE) were trained on larger samples and incorporate data on mortality (31, 32), the pace of aging (33), and health behaviors such as cigarette smoking (32). Thus, the newer clocks tend to be more strongly associated with age-related traits and diseases. Previous studies have found that higher PhenoAge acceleration (PhenoAA) and DunedinPACE were associated with lower HDL-C and higher TG (31, 34), and higher HannumAge acceleration (HannumAA) was associated with lower HDL-C (35, 36) and higher TC (37). However, the generalization of these findings is unknown because these studies are demographically limited (e.g., comprised of a single race/ethnicity or sex) or did not adjust for important demographic and behavioral factors such as educational attainment and cigarette smoking. In this study, we examined the associations between these five measures of epigenetic age acceleration and blood lipids in a representative sample of older adults from multiple racial/ethnic groups in the U.S.. We also tested whether the associations differ by demographic factors including age, sex, and educational attainment. Characterizing the associations between biological aging and blood lipid levels may help guide the early prevention and treatment efforts for dyslipidemia.

## Methods

### Study sample

The Health and Retirement Study (HRS) is a U.S. nationally representative, longitudinal study of adults aged over 50 years and their spouses from multiple racial/ethnic groups (38). The study was first conducted in 1992 to collect data on health, financial, and economic factors at both the individual and community levels, with a follow-up every two years. An ancillary study of HRS, the 2016 Venous Blood Study (VBS), collected DNA methylation data and biomarkers such as blood lipid measures, fasting glucose, and inflammatory markers (39). DNA methylation measurement was performed on a subset of respondents that were randomly selected from the 2016 VBS study. Participants aged 55 and older were assigned a VBS DNA methylation weight, which adjusted for the differential probabilities of participation for age-eligible respondents at the time of the VBS (i.e., those born before 1960) (40, 41). By incorporating the VBS DNA methylation weight into analysis, the sample is representative of U.S. adults aged 55 years or older. After excluding participants missing all four blood lipid measures (n = 10), any of the covariates (n = 55), or VBS DNA methylation weight (n = 140), a total of 3,813 participants remained in the study. All participants provided written informed consent before participation.

### Lipid measures

Participants were encouraged but not required to fast before the blood draw. TC and TG were both measured in serum using a Roche Cobas 6000 Chemistry Analyzer (Roche Diagnostics Corporation) (42). Serum HDL-C was measured using the Roche HDL-Cholesterol 3rd generation direct method at Roche Diagnostics (Indianapolis, IN) (42). LDL-C was only calculated for participants whose TG levels were no higher than 400 mg/dL using Friedewald’s formula: LDL (mg/dL) = TC– HDL-C – (TG/5.0) (43). HDL-C and TG were natural log-transformed prior to analysis. For each blood lipid measure, outliers of more than 4 standard deviations (SD) from the mean were excluded from the analyses. Participants indicated whether they fasted or took lipid-lowering medication prior to the blood draw.

### DNA methylation and epigenetic age acceleration

#### DNA methylation

DNA methylation was measured in whole blood samples using the Infinium MethylationEPIC BeadChip for 4,104 participants. Samples were randomized across plates by key demographic variables including age, cohort, sex, education, and race/ethnicity (40). Data preprocessing and quality control were performed using the *minfi* package in the R software. Probes with a detection P-value > 0.01 were removed. Samples were removed if they had > 5% missing probes or mismatched sex. The final DNA methylation sample consists of 4,018 participants. Prior to the epigenetic clock estimation, missing beta methylation values were imputed to the mean beta methylation value of the given probe for all samples (40).

#### Epigenetic age acceleration

HorvathAge and HannumAge use CpG sites associated with chronological age. HorvathAge, calculated using 353 CpG sites, was developed in samples of multiple tissues and cell types (29). HannumAge, calculated using 71 CpG sites, was developed in whole-blood samples (30). PhenoAge, calculated using 513 CpG sites, was developed to capture chronological aging and 9 aging biomarkers in whole blood samples (31). GrimAge, which includes 1,030 CpG sites, was developed to capture cigarette smoking pack-years, a known risk for mortality, and other 7 mortality-associated plasma proteins in whole-blood samples (32). DunedinPACE, a DNA methylation biomarker that uses 173 CpG sites, was developed in blood samples taken longitudinally to capture individual variation in the *rate* of biological aging. All clocks in this study have been publicly released for HRS (40) except for DundeinPACE which we estimated using the DunedinPACE projector provided by Belsky et al. (33).

HorvathAge acceleration (HorvathAA), HannumAA, PhenoAA, GrimAge acceleration (GrimAA), and DunedinPACE were evaluated in this study. All measures of DNA methylation age acceleration (DNAmAA) except for DunedinPACE were estimated by calculating the residuals obtained from regressing each epigenetic age measure on the participants’ chronological age, with a one-unit change representing a one-year increase in biological aging compared to chronological age. The interpretation of DunedinPACE is fundamentally different, with each unit representing a rate of one-year of biological aging per each year of chronological aging. For each measure of DNAmAA, outliers of more than 4 SD from the mean were windsorized.

### Covariates

Age at methylation measurement, sex, self-reported race/ethnicity (non-Hispanic White, non-Hispanic Black, Hispanic, all other groups), body mass index (BMI), smoking status (never smoker, former smoker, current smoker), fasting status, lipid-lowering medication use, and educational attainment (less than high school degree, high school degree or equivalent, college degree and above) were collected at the time of the blood draw.

### Statistical analysis

Pearson correlations were calculated among the epigenetic clocks and age acceleration measures. We used weighted linear regression to examine the associations between demographic factors including age, sex, educational attainment (3-level variable) as independent variables and each blood lipid measure, adjusting for race/ethnicity, fasting status at blood draw, and lipid-lowering medication use, BMI, and smoking status. We then used weighted linear regression models to explore the associations between each measure of epigenetic age acceleration (independent variable) and each of the blood lipid measures (dependent variable). Model 1 adjusted for age, sex, race/ethnicity, fasting status at blood draw, and lipid-lowering medication use. Model 2 further adjusted for BMI, smoking status, and educational attainment (3-level variable). *P* < 0.05 was considered significant for correlations and weighted linear models. We next conducted the same analysis within each racial/ethnic group separately (Model 2). Since fasting status greatly influences TG (44), and because lipid-lowering medications can have a large impact on lipid profiles as well as DNA methylation (45), we also conducted sensitivity analyses by examining associations (Model 2) after stratifying our sample by fasting status (yes/no) and lipid-lowering medication use (yes/no).

Interactions were only tested for associations in which both the epigenetic age acceleration and the demographic factor were associated with the corresponding blood lipid in Model 2 at *P* < 0.05. We introduced multiplicative terms for each demographic factor (age, sex, educational attainment) and epigenetic age acceleration to examine whether the associations differed by these demographic factors. Age was centered for the interaction analyses. To better capture interactions with educational attainment, we examined potential interactions using two dichotomous variables separately: (1) less than high school degree vs. high school degree and above, and (2) less than college degree vs. college degree and above. For significant interactions (*P* < 0.05 for the interaction term), we created plots to illustrate the associations between the epigenetic age acceleration measures and blood lipids at each level of the categorical factors (for sex and educational attainment). For age, we presented the associations at the 25th percentile (62 years old) and 75th percentile (77 years old) of the sample. All statistical analyses were conducted in R software (version 4.3.1) using the survey, emmeans, and siPlot packages.

## Results

The mean age of the participants (N = 3,813) was 70.0 years, and the majority were female (57.7%, Table 1). Participants self-identified as non-Hispanic White (67.2%), non-Hispanic Black (16.3%), Hispanic (13.3%), or some other group (3.1%). Over half of the participants had at least a high school degree (59.1%) and 24.3% had at least a college degree. A total of 44.2% were non-smokers, 44.7% were former smokers, and 11.1% were current smokers. The mean BMI was 28.9 kg/m^2^. Nearly half of the participants were using lipid-lowering medications (49.0%), and 66.4% of the participants reported fasting at the time of the blood draw.

Correlations among the epigenetic clocks ranged from 0.64 to 0.77 (**Supplemental Table 1A**), and correlations among DNAmAA measures ranged from − 0.03 to 0.60, with HorvathAA having particularly weak correlations with more recently developed DNAmAA measures (**Supplemental Table 1B**). All correlations had P-values < 0.05 except for HorvathAA and DunedinPACE. Older age was associated with lower levels of all lipid measures (P < 0.05) except for HDL-C (**Supplemental Table 2**). Being a female was associated with higher levels of TC, HDL-C, and LDL-C compared to males. Finally, compared with those who did not have a high school degree, having a high school degree or equivalent was associated with higher HDL-C, and having a college degree and above was associated with higher HDL-C and lower TG.

Higher DNAmAA was associated with lower levels of TC, HDL-C, and LDL-C, and higher levels of TG in Model 1 (*P* < 0.05; Table 2) for all DNAmAA measures except for HorvathAA. After further adjustment for BMI, smoking status, and educational attainment (Model 2), GrimAA and DunedinPACE remained associated with all lipid measures. All other associations remained significant although most had slightly attenuated effect estimates, except for PhenoAA with TC and HannumAA with LDL-C and TG, which were no longer significant. Among all significant associations in Model 2, DunedinPACE had the most significant associations with all blood lipids, and GrimAA had the second most significant associations. HannumAA and PhenoAA shared similar but smaller estimates. Specifically, a one-unit increase in DunedinPACE was associated with decreases of 38.74 mg/dL, 20.55 mg/dL, and 27.87 mg/dL in TC, HDL-C, and LDL-C, respectively, and an increase of 73.17 mg/dL in TG. Additionally, a one-year increase in GrimAA was associated with decreases of 0.66, 0.51, and 0.62 mg/dL in TC, HDL-C, and LDL-C, respectively, and an increase of 1.75 mg/dL in TG. Because HDL-C and TG were natural log-transformed prior to analyses, these effect size estimates, and those reported subsequently, correspond to a person with the mean blood lipid level in the sample (i.e., HDL-C at 57.0 mg/dL or TG at 144.8 mg/dL). Most of the associations remained significant in non-Hispanic White and non-Hispanic Black samples, with similar effect estimates. Few associations were detected in Hispanics (**Supplemental Table 3**).

We further stratified the sample by fasting status (**Supplemental Table 4**) and lipid-lowering medication use (**Supplemental Table 5**), respectively, in Model 2 to see whether the associations between epigenetic age acceleration and lipid levels vary across groups. Associations between DNAmAA and blood lipids were detected in participants who both fasted and did not fast, except for LDL-C, which was only detected in participants who fasted. We also detected associations mainly in participants who did not use lipid-lowering medication at the time of the blood draw. Overall, DunedinPACE had the most consistent associations among the DNAmAA measures, as it was associated with all lipids across all groups except for LDL-C in non-fasting participants.

**Figure 1:**
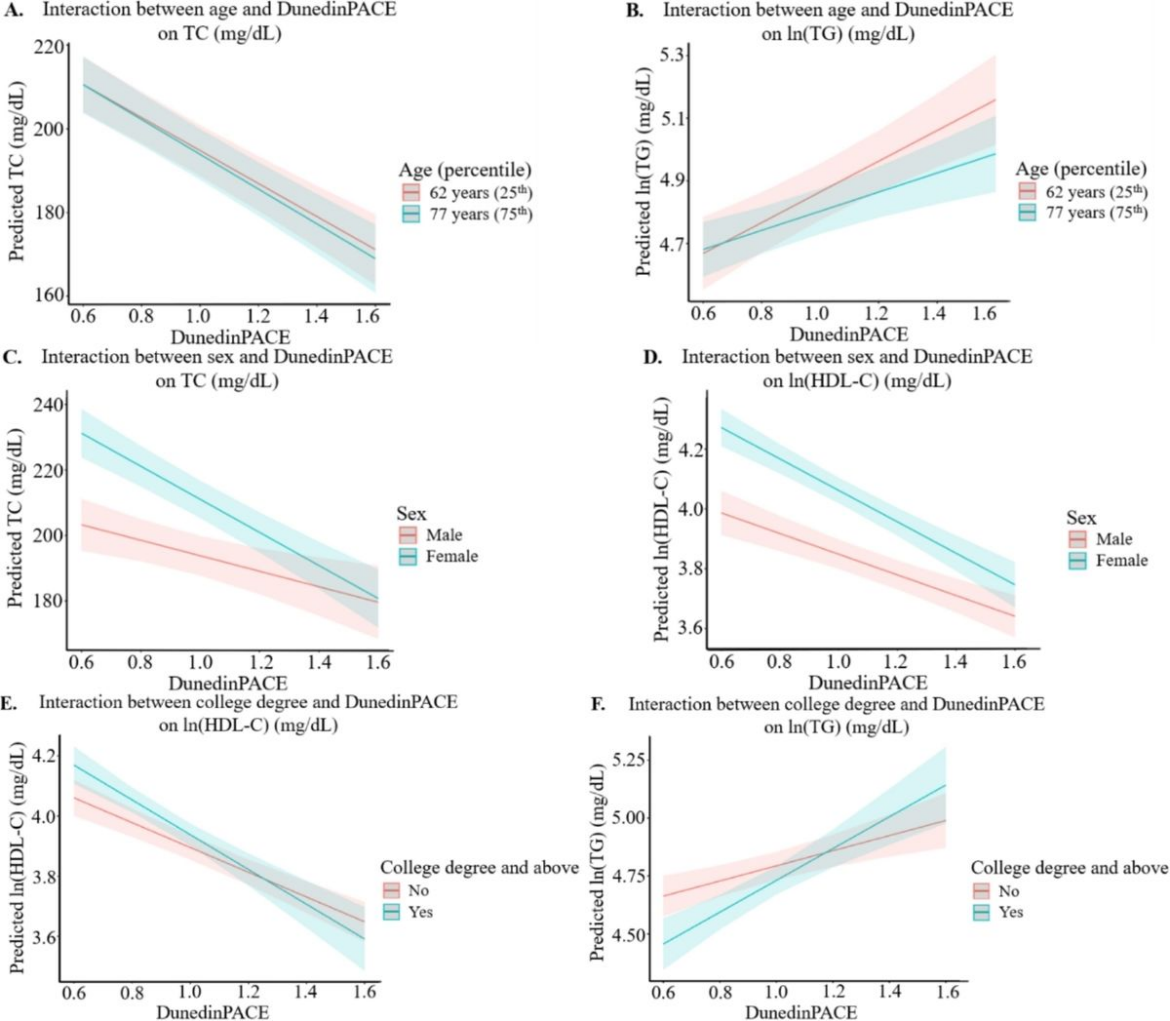
Plots of predicted blood lipid levels by DunedinPACE at the 25^th^ (62 years) and 75^th^ percentile (77 years) of age (A,B), sex (C,D) and college degree (E,F). TC, total cholesterol; HDL-C, high-density lipoprotein; TG, triglycerides Interaction model: blood lipid level ~ epigenetic age acceleration + age at methylation measurement + sex + race/ethnicity + fasting status + lipid-lowering medication use + body mass index + smoking status + high school degree or equivalent + college degree and above + DunedinPACE × demographic factor Only interactions with DunedinPACE and demographic factors with P_interaction_ < 0.05 in the interaction model are shown. Age was scale and centered for the interaction analysis. The line and corresponding confidence intervals represent the predicted blood lipid level at the corresponding value of DunedinPACE.

Next, to understand whether associations between DNAmAA and blood lipid levels are modified by demographic factors, we introduced multiplicative terms into Model 2 (Table 3, Fig. 1, **Supplemental Fig. 1**). Six interactions between DunedinPACE and demographic factors on blood lipids were detected (Fig. 1). For TC, a one-unit increase in DunedinPACE was associated with a decrease of 29.99 mg/dL in younger participants (aged 62 years, 25th percentile) vs. a 49.76 mg/dL in older participants (aged 77 years, 75th percentile) (Fig. 1.A). For TG, a one-unit increase in DunedinPACE was associated with an increase of 42.29 vs. 97.10 mg/dL in younger vs. older participants (Fig. 1.B). In addition, the inverse associations between DunedinPACE and both TC and HDL-C were stronger in females compared with males, as a one-unit increase in DunedinPACE was associated with a decrease of 50.51 vs. 23.65 mg/dL in TC (Fig. 1.C) and a decrease of 23.32 vs. 16.67 mg/dL in HDL-C in females vs. males (Fig. 1.D). Finally, for those with a college degree vs. without, a one-unit increase in DunedinPACE was associated with a decrease of 19.29 vs. 25.05 mg/dL in HDL-C (Fig. 1.E) and an increase of 55.81 vs. 142.45 mg/dL in TG (Fig. 1.F). The significant interactions between GrimAA and PhenoAA and demographic factors on blood lipids showed similar trends (**Supplemental Fig. 1.A-C**) However, interactions between HannumAA and educational attainment on TG and HDL-C showed different patterns of association than DunedinPACE (**Supplemental Fig. 1.D-E**).

## Discussion

In this study, greater epigenetic age acceleration was associated with lower TC, HDL-C, LDL-C, and higher TG, with stronger associations detected with GrimAA and DunedinPACE. The associations were mostly consistent across racial/ethnic groups. Moreover, these associations were stronger in participants who fasted prior to the blood draw and who did not take lipid-lowering medication, particularly for LDL-C. We detected the largest number of demographic factor interactions with DunedinPACE, in which the associations tended to be stronger among younger participants, females, and those with higher educational attainment. To our knowledge, this is the first study examining the associations between epigenetic age acceleration and blood lipids, as well as interactions with demographic factors, in a multi-racial/ethnic representative cohort of older U.S. adults.

We found that higher epigenetic age acceleration was associated with lower levels of TC, HDL-C, and LDL-C, as well as higher levels of TG, except for HorvathAA. These results align with the previous findings in samples consisting of a single racial/ethnic group. For instance, higher GrimAA was associated with elevated TG levels in older African Americans (mean age = 57 years) in the Genetics Epidemiology Network of Arteriopathy (GENOA) study (46). Moreover, Higher HannumAA and DunedinPACE were associated with lower HDL-C and higher TG in middle-aged non-Hispanic Whites and Asians from the Genetics of Lipid Lowering Drugs and Diet Network (GOLDN) study (35) and the Taiwan Biobank (34), respectively. By using a multi-racial/ethnic representative cohort of older U.S. adults, our results further demonstrated the strong associations between biological aging and lipids. However, the inverse associations we detected between epigenetic age acceleration and both TC and LDL-C did not align with our hypothesis that higher age acceleration measures are associated with higher TC and LDL-C levels, given the adverse effects of these lipids on health (47, 48). Our finding might be explained by the non-linear relationships between age and TC/LDL-C, as these lipids tend to decrease more rapidly at older ages (49, 50) or the lipid-lowering medication use among those with a diagnosis of high cholesterol levels. Previous studies of the associations between epigenetic age acceleration and TC also presented mixed findings. For example, an inverse association was detected between PhenoAA and TC in the National Health and Nutrition Examination Survey (NHANES) (31), while a positive association between HannumAA and TC was found from the GENOA (46). The inconsistency in findings may stem from racial/ethnic differences, age variations, and inclusion criteria in the samples. NHANES, as a U.S. representative cohort, consists of participants of all ages (31), while GENOA consists of older African American sibships enriched for hypertension (46). The average age of our sample (70 years) may have led to selection bias, in which our older respondents are healthier, resulting in lower TC levels. The inverse associations between epigenetic age acceleration and LDL-C we detected were also consistent with previous findings (34, 46).

GrimAA and DunedinPACE exhibited the strongest associations with all blood lipids, while almost no significant associations were detected with earlier developed clocks. The correlation among epigenetic age measures were relatively high, but correlations among the epigenetic age acceleration measures were much weaker, with HorvathAA showing particularly weak correlations with acceleration measures from more recently developed clocks. Earlier clocks such as HorvathAge and HannumAge were trained on chronological age (29, 30), whereas later clocks including PhenoAge, GrimAge, and DunedinPACE incorporate lifespan mortalities, morbidities, and health behaviors such as smoking (31–33). GrimAge incorporates CpGs that capture cigarette smoking pack-years, known as an independent risk factor for unfavorable lipid profiles (32, 51). While we adjusted for smoking as a three-category variable, it may not fully eliminate the effects of smoking. Therefore, the stronger associations we observed between GrimAA and lipids could be partially attributed to the residual confounding effects of smoking. DunedinPACE has been associated with social disadvantage and can better predict future functional limitations and chronic disease morbidity than other clocks (33).

In the sensitivity analysis, we observed stronger associations, particularly for LDL-C, in participants who fasted prior to the blood draw and those not using lipid-lowering medication. Fasting can greatly impact TG levels (44), as an observational study suggests that while fasting and non-fasting TC and HDL-C exhibit similar performance, non-fasting TG levels may be substantially higher compared to fasting (52). Consequently, LDL-C levels might be underestimated when using non-fasting TG measures for calculation from the Friedewald formula (43). This could help explain why epigenetic age acceleration was only associated with LDL-C in fasting samples in our study. Similarly, the use of lipid-lowering medications can significantly alter lipid profiles, with notable decreases in TC and LDL-C observed in statin users (45). This supports our observation that associations between epigenetic age and lipids are stronger in participants not using lipid-lowering medication, suggesting that lipid-lowering medications might mask the relationships between epigenetic age acceleration and blood lipids.

In this study, the inverse association between DunedinPACE and TC was slightly stronger in older participants, and this might be explained by faster decrease in TC levels in older adults (49). On the other hand, the positive association between DunedinPACE and TG was stronger in younger participants. Animal models have demonstrated the non-linear relationship of age and TG, with TG levels rising rapidly initially but beginning to decrease in older ages (53). Findings from the Prospective Cardiovascular Müinster (PROCAM) study found that TG levels in males increased until 35 years old and began to decrease after age 55 (54). However, few studies have examined the effects of age on TG levels in longitudinal population studies, limiting our understanding of the associations between epigenetic age acceleration and TG in adults as they grow older. Further studies are required to fill this gap.

The associations between DunedinPACE and both TC and HDL-C were negative in both sexes, although stronger in females. The rapid decline in TC and HDL-C levels among females as epigenetic age acceleration increases may be attributed to menopause, given that postmenopausal females had less favorable lipid profiles, including lower TC and HDL-C levels, compared with premenopausal females (55, 56). Epigenetic age acceleration has also been associated with age at menopause in an early study (57). We also found that being female was associated with higher levels of HDL-C compared to being male without adjusting for epigenetic age acceleration, which aligns with prior studies (50, 55). Unhealthy behaviors such as smoking, alcohol consumption, and lack of exercise are associated with dyslipidemia, obesity (58), and an elevated risk of coronary heart disease (59), with men being more prone to engaging in these behaviors. For example, less than half of females were current or former smokers compared to almost 65% of males in our sample.

Finally, the associations between higher DunedinPACE and both lower HDL-C and higher TG were both stronger among those with a higher educational attainment. Studies also consistently reported that greater educational attainment and high levels of socioeconomic status are associated with elevated HDL-C levels (60–62), lower prevalence of hypertriglyceridemia (63), and lower epigenetic age (64, 65). Moreover, epigenetic age acceleration has been inversely associated with HDL-C and positively associated with TG levels (46, 66). Therefore, the participants in our study who had a college degree might have had healthier HDL-C and TG profiles with lower epigenetic age acceleration, but the effects were attenuated with accelerated biological aging.

This work has many strengths. We were able to examine the associations between epigenetic age acceleration and blood lipids in a nationally representative and multi-racial/ethnic cohort of older U.S. adults. Our success in replicating the results from previous studies consisting of smaller and more homogeneous samples also implies the solid role of biological aging in blood lipid levels across racial/ethnic and demographic groups. In addition, through sensitivity analysis, we were able to examine the associations between epigenetic aging and blood lipids without the influence of fasting status or lipid-lowering medications. Lastly, the interaction analyses shed light on the modification roles of demographic factors on the relationships between epigenetic aging and blood lipids. This study also has limitations. First, we cannot infer a causal relationship given that the methylation and blood lipids were measured contemporaneously. Second, the VBS was an optional sub-study for the HRS participants, which could have introduced participation bias. However, by incorporating sample weights, we expect the bias to be minimal. Third, because the epigenome is influenced by the environment, there may be other factors that influenced methylation at the time of collection which cannot be fully evaluated.

## Conclusions

In summary, we have found evidence of associations between epigenetic age acceleration, a powerful biological aging marker, and blood lipid levels, with stronger associations detected with GrimAA and DunedinPACE. Sensitivity analysis highlighted stronger associations in participants who fasted or did not take lipid-lowering medication, particularly for LDL-C. Furthermore, associations between DunedinPACE and blood lipids were stronger in younger participants, females, and individuals with higher educational attainment. The observed association of higher epigenetic age acceleration with lower TC may be influenced by sample limitations or the non-linear relationship between age and TC. Future studies and further exploration of the potential clinical implications of epigenetic age acceleration on blood lipids and other health outcomes are warranted. Overall, our findings contribute to an enhanced understanding of the potential mechanisms linking biological aging and lipid profiles.

## Figures and Tables

**Table 1:** Descriptive characteristics of the Health and Retirement Study participants (N = 3,813)

Characteristic	Mean (SD) or N (%)
Age (years)	70.0 (9.3)
Female sex	2201 (57.7%)
Race/ethnicity (self-reported)	
Non-Hispanic White	2564 (67.2%)
Non-Hispanic Black	623 (16.3%)
Hispanic	509 (13.3%)
All other groups	117 (3.1%)
Educational attainment	
Less than high school degree	633 (16.6%)
High school degree or equivalent	2254 (59.1%)
College degree and above	926 (24.3%)
BMI (kg/m^2^)	28.9 (6.3)
Smoking status	
Never smoker	1685 (44.2%)
Former smoker	1704 (44.7%)
Current smoker	424 (11.1 %)
Lipid-lowering medication use	1869 (49.0%)
Fasting at time of blood draw	2531 (66.4%)
**Blood lipids**	
Total cholesterol (mg/dL) (n = 3,811)	187.6 (41.6)
HDL-C (mg/dL)^a^	57.0 (18.8)
LDL-C (mg/dL) (n = 3,731)	101.7 (35.0)
Triglycerides (mg/dL) (n = 3,806)^a^	144.8 (84.0)
**Epigenetic age acceleration**	
HorvathAA (years)	0.017 (6.3)
HannumAA (years)	−0.018 (5.1)
PhenoAA (years)	−0.036 (6.8)
GrimAA (years)	0.009 (4.7)
DunedinPACE	1.038 (0.1)

SD, standard deviation; TC, total cholesterol; HDL-C, high-density lipoprotein; LDL-C, low-density lipoprotein; TG, triglycerides; HorvathAA, HorvathAge acceleration; HannumAA, HannumAge acceleration; PhenoAA, LevineAge (PhenoAge) acceleration; GrimAA, GrimAge acceleration; BMI, body mass index

a HDL-C and triglycerides were natural-log transformed prior to analysis

**Table 2:** Associations between epigenetic age acceleration and blood lipids

	Model 1^a^			Model 2^b^		
	β^c^	SE	P	β^c^	SE	P
**TC (n = 3,811)**						
HorvathAA	0.141	0.109	0.200	0.181	0.110	0.107
HannumAA	−0.322	0.113	**0.007**	−0.261	0.112	**0.024**
PhenoAA	−0.254	0.105	**0.019**	−0.195	0.103	0.065
GrimAA	−0.552	0.149	**0.001**	−0.656	0.191	0.001
DunedinPACE	−41.90	4.051	**8.35×10** ^**− 14**^	−38.74	4.417	**3.95×10** ^**− 11**^
**ln(HDL-C) (n = 3,813)**						
HorvathAA	−0.0001	0.001	0.892	0.001	0.001	0.533
HannumAA	−0.004	0.001	**0.002**	−0.002	0.001	**0.050**
PhenoAA	−0.004	0.001	**8.49×10** ^**− 5**^	−0.002	0.001	**0.017**
GrimAA	−0.011	0.001	**3.84×10** ^**− 10**^	−0.009	0.002	**2.44×10** ^**− 5**^
DunedinPACE	−0.633	0.041	**3.56×10** ^**− 20**^	−0.447	0.044	**4.08×10** ^**− 13**^
**LDL-C (n = 3,731)**						
HorvathAA	0.069	0.089	0.442	0.084	0.089	0.352
HannumAA	−0.220	0.106	**0.043**	−0.202	0.106	0.064
PhenoAA	−0.239	0.091	**0.011**	−0.220	0.090	**0.018**
GrimAA	−0.501	0.142	**0.001**	−0.619	0.166	**0.001**
DunedinPACE	−26.83	4.471	**2.50×10** ^**−7**^	−27.87	4.882	**9.69×10** ^**−7**^
**ln(TG) (n = 3,806)**						
HorvathAA	0.002	0.002	0.218	0.001	0.002	0.378
HannumAA	0.004	0.002	**0.042**	0.002	0.002	0.241
PhenoAA	0.006	0.002	**4.76×10** ^**− 4**^	0.004	0.002	**0.012**
GrimAA	0.014	0.002	**2.90×10** ^**− 7**^	0.012	0.003	**3.89×10** ^**− 5**^
DunedinPACE	0.633	0.064	**4.19×10** ^**− 13**^	0.409	0.073	**1.35×10** ^**− 6**^

TC, total cholesterol; HDL-C, high-density lipoprotein; LDL-C, low-density lipoprotein; TG, triglycerides; HorvathAA, HorvathAge acceleration; HannumAA, HannumAge acceleration; PhenoAA, PhenoAge acceleration; GrimAA, GrimAge acceleration

a Model 1: blood lipid level = epigenetic age acceleration + age at methylation measurement + sex + race/ethnicity + fasting status + lipid-lowering medication use

b Model 2: Model 1 + body mass index + smoking status + educational attainment (less than high school degree, high school degree or equivalent, college degree and above)

c β corresponds to the change in blood lipid level associated with a 1-unit increase in the measure of epigenetic age acceleration.

P-value < 0.05 in bold

**Table 3:** Interactions between epigenetic age acceleration and demographic factors on blood lipid levels

Multiplicative term	Lipid	DNAmAA	β_NAmAA_	P_DNAmAA_	β_Demo_	P_Demo_	β_interaction_	P_interaction_
DNAmAA×Age^a^	TC	GrimAA	−0.755	**2.33×10** ^**−4**^	−0.727	**2.51×10** ^**−13**^	−0.051	**0.004**
		DunedinPACE	−40.57	**3.25×10** ^**−12**^	0.760	0.190	−1.318	**0.018**
	ln(TG)	GrimAA	0.011	**4.84×10** ^**−5**^	−0.004	0.002	**-4.94×10** ^**−4**^	**0.037**
		DunedinPACE	0.392	**2.05×10** ^**−6**^	0.008	0.173	−0.012	**0.033**
DNAmAA×Sex^b^	TC	DunedinPACE	−23.65	**0.002**	44.10	**5.62×10** ^**−5**^	−26.86	**0.007**
	ln(HDL-C)	PhenoAA	−0.0005	0.737	0.221	**6.10×10** ^**−23**^	−0.003	**0.037**
		GrimAA	−0.005	**0.033**	0.197	**1.14×10** ^**−19**^	−0.008	**0.007**
		DunedinPACE	−0.346	**8.76×10** ^**−8**^	0.393	**2.34×10** ^**−7**^	−0.180	**0.005**
DNAmAA×High school degree^c^	ln(HDL-C)	HannumAA	−0.009	**0.003**	0.072	**1.14×10** ^**−4**^	0.007	**0.018**
DNAmAA×College degree^d^	ln(HDL-C)	DunedinPACE	−0.413	**7.81×10** ^**−11**^	0.209	**0.018**	−0.166	**0.049**
	ln(TG)	HannumAA	0.005	0.057	−0.083	**0.002**	−0.008	**0.036**
		DunedinPACE	0.326	**2.46×10** ^**−4**^	−0.422	**0.004**	0.359	**0.011**

TC, total cholesterol; HDL-C, high-density lipoprotein; TG, triglycerides; DNAmAA: epigenetic age acceleration; HannumAA, HannumAge acceleration; PhenoAA, LevineAge (PhenoAge) acceleration; GrimAA, GrimAge acceleration; Demo, demographic factors

Model: blood lipid level ~ epigenetic age acceleration + age at methylation measurement + sex + race/ethnicity + fasting status + lipid-lowering medication use + body mass index + smoking status + high school degree or equivalent + college degree and above + epigenetic age acceleration × demographic factor

We only tested for an interaction when both the DNAmAA and the demographic factor were associated with blood lipids in Model 2.

Only significant interactions between epigenetic age acceleration and demographic factors (P_Interaction_<0.05) were included in this table.

DNAmAA effect sizes (β_DNAmAA_) correspond to the change in blood lipid level associated with a 1-unit increase in DNAmAA. Demographic factor effect sizes (β_Demo_) correspond to the change in blood lipid level associated with a 1-unit increase in age or with the non-reference level for sex or educational attainment. Interaction effect sizes (β_Interaction_) correspond to the change in effect of β_DNAmAA_ on blood lipids for each 1-unit increase (or level) of the demographic factor.

a Age was centered in this analysis.

b Reference group: male

c Reference group: less than high school degree

d Reference group: less than college degree

P-value < 0.05 in bold

## Data Availability

HRS epigenetic clocks, blood lipid levels, and survey data are publicly available from the HRS website at https://hrs.isr.umich.edu/data-products. The DunedinPACE clock can be calculated with HRS epigenetic data accessible from the National Institute on Aging Genetics of Alzheimer’s Disease Data Storage Site (NIAGADS), accession number: ng00153, using the codes provided at https://github.com/danbelsky/DunedinPACE/tree/main.
